# ^131^I-labeled engineered hybrid cell membrane biomimetic nanocarrier targets gastric cancer and enhances radioimmunotherapy

**DOI:** 10.1016/j.mtbio.2026.103533

**Published:** 2026-08-04

**Authors:** Chuanqiang Zhang, Hua Chen, Zheng Yuan, Anqi Dong, Shu Liu, Lin Hu, Kai Yang, Jin Zhou

**Affiliations:** a Department of General Surgery, The First Affiliated Hospital of Soochow University, Suzhou, Jiangsu, 215006, People's Republic of China; b Department of General Surgery, The Affiliated Jiangsu Shengze Hospital of Nanjing Medical University, Suzhou, Jiangsu, 215228, People's Republic of China; c State Key Laboratory of Radiation Medicine and Protection, School of Radiation Medicine and Protection & School for Radiological and Interdisciplinary Sciences (RAD-X), Collaborative Innovation Center of Radiation Medicine of Jiangsu Higher Education Institutions, Soochow University, Suzhou, Jiangsu, 215123, People's Republic of China

**Keywords:** Radiolabeling, Hybrid cell membrane, Immunotherapy, Gastric cancer treatment

## Abstract

Achieving efficient radiolabeling and targeted delivery is essential to maximize therapeutic efficacy and reduce systemic toxicity. Herein, we report a novel radionuclide-delivery platform based on hybrid membranes derived from fusing liposome with statherin engineered macrophage membranes and gastric cancer cell (MFC) membranes. The obtained ^131^I-lipo@[RAW-MFC] nanoparticles (^131^I-Lip^RM^) exhibit high-yield, stable radiolabeling and homologous tumor targeting. Using a gastric cancer model, we assess the biodistribution and therapeutic potential of ^131^I-Lip^RM^ alone and combine with anti-PD-L1 therapy. ^131^I-Lip^RM^ show favorable pharmacokinetics and efficient tumor localization, inducing significant tumor cell damage and growth suppression. Furthermore, the combination of ^131^I-Lip^RM^ with anti-PD-L1 therapy has synergistically enhanced T cell-mediated immune responses, promoted CD8^+^ T cell infiltration, and prolonged survival relative to monotherapies. These findings demonstrate that the ^131^I-Lip^RM^ platform enables precise radionuclide delivery and potentiates antitumor immunity through PD-L1 blockade, presenting a promising avenue for radio-immunotherapy.

## Graphical abstract

**Figure:**
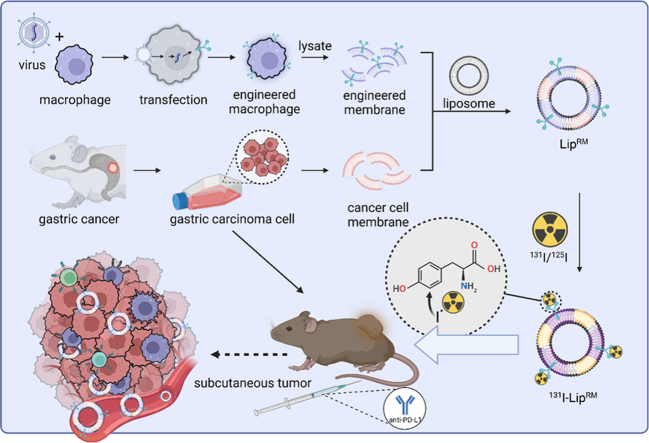

## 1 Introduction

Gastric cancer (GC) remains a formidable global health challenge and the third leading cause of cancer-related mortality, largely due to its frequent diagnosis at advanced stages [[1], [2], [3], [4]]. The aggressiveness and heterogeneity of GC often render conventional targeted therapies ineffective [5]. Furthermore, the immunosuppressive GC microenvironment severely compromises endogenous antitumor immune responses [[6], [7], [8], [9]]. Consequently, there is an urgent need for innovative strategies that enhance the therapeutic efficiency of advanced GC.

Radiotherapy, particularly radionuclide therapy, has garnered widespread attention in recent years for its ability to induce immunogenic cell death (ICD) in tumors, release cancer associated antigens, and subsequently activate anti-tumor immune responses [[10], [11], [12], [13], [14]]. However, their clinical potential is constrained by poor biodistribution, leading to off-target accumulation and radiation-induced damage in normal organs [[15], [16], [17], [18], [19]]. To address these limitations, cell membrane-camouflaged nanotechnology utilizing hybrid membranes has emerged as a powerful strategy to enhance targeting specificity by integrating complementary biological mechanisms [[20], [21], [22], [23], [24]]. This approach not only enhances biocompatibility and immune evasion, but also improves targeting capability through membrane-specific recognition molecules for active targeting [[25], [26], [27], [28]]. Among various options, macrophage membranes have emerged as ideal candidates due to their inherent tropism toward inflammatory and tumoral microenvironments [[29], [30], [31], [32]]. By fusing macrophage membranes with gastric cancer cell membranes, we could combine the inflammatory tropism of macrophages with the homologous targeting ability of cancer cells. This dual-membrane hybridization exploited specific receptors and adhesion molecules to facilitate precise nanoparticle accumulation within tumors. Furthermore, membrane engineering strategies enabled the over-expression or incorporation of functional proteins on the cell membrane surface, which further enhanced the stable labeling of radionuclides. For instance, the incorporation of tyrosine-rich proteins into macrophage membranes allowed for efficient and stable radiolabelling with iodine isotopes, significantly improving radionuclide retention and therapeutic efficacy. Such bioengineering approaches has provided versatile platforms for targeted radionuclide delivery, drug conjugation, and immune modulation, ultimately advancing the development of multifunctional biomimetic nanocarriers for cancer theranostics [[33], [34], [35], [36], [37]].

Recent progress in radionuclide therapy has demonstrated substantial promise for gastrointestinal malignancies. The strongest clinical evidence stems from gastroenteropancreatic neuroendocrine tumors (GEP-NETs), in which somatostatin receptor (SSTR)-targeted peptide receptor radionuclide therapy using ^177^Lu-DOTATATE has become a standard treatment option, further reinforced by the phase III NETTER-2 trial. Beyond GEP-NETs, emerging modalities have broadened the therapeutic landscape, including FAP-targeted ^177^Lu-FAP-2286, CEA-targeted ^177^Lu-nanobody therapy for colorectal cancer, CLDN18.2-targeted [^131^I]I-zolbetuximab for gastric cancer, and ^90^Y radioembolization for liver-predominant gastrointestinal tumors. Despite these advances, current radionuclide approaches face major limitations, such as heterogeneous target expression, poor tumor retention, off-target radiation exposure, slow pharmacokinetics of antibody-based agents, and restricted efficacy to selected tumor types or anatomical sites. These challenges collectively emphasize the need for novel delivery systems that offer improved labeling stability, enhanced tumor accumulation, and favorable biosafety profiles. To address this, we designed a hybrid membrane-biomimetic nanocarrier that integrates tyrosine-enriched radioiodination sites, macrophage-associated tumor tropism, and homologous gastric cancer cell membrane-mediated targeting, providing a rational platform for augmented ^131^I delivery and radioimmunotherapy in gastric cancer.

**Scheme 1:**
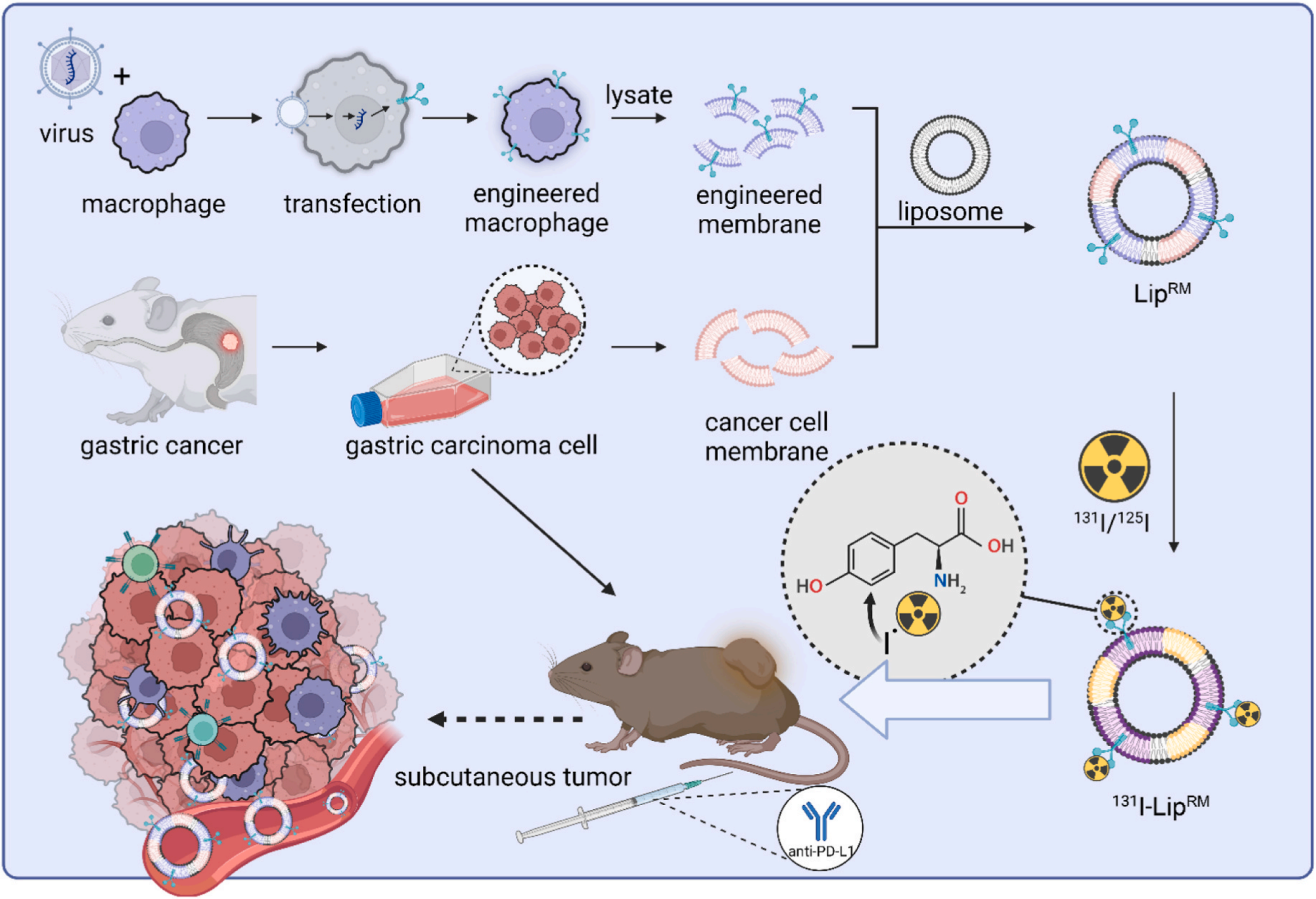
Schematic illustration of the construction and application of ^131^I-Lip^RM^.

In this study, we collected the macrophage membranes rich in tyrosine-containing proteins and gastric cancer cell membranes (MFC) and then fused them with the liposomes to construct biomimetic nanoparticles with dual-membrane functionality. This hybrid membrane nanoparticles (Lip^RM^) was subsequently labeled with the radionuclide ^131^I, enabling efficient and targeted delivery to tumor sites (Scheme 1). Leveraging the homologous targeting, ^131^I-Lip^RM^ could effectively accumulate at tumor sites and continuously induce tumor cell death. Beyond inducing direct apoptosis, the ^131^I-Lip^RM^ also triggered ICD, characterized by the release of danger-associated molecular patterns (DAMPs), such as ATP, HMGB1, and calreticulin (CRT), which further enhanced tumor antigen recognition and immune activation. To further amplify these immunostimulatory effects, we combined the ^131^I-Lip^RM^ with anti-PD-L1 checkpoint blockade. This combination aimed to rescue T cell function and promoted systemic antitumor immunity by synergizing with radiation-induced ICD. Collectively, this study presents a hybrid membrane-based biomimetic platform for efficient ^131^I delivery and immune activation, offering a promising strategy for synergistic radioimmunotherapy and a potential pathway for the clinical translation of integrated gastric cancer treatments.

## 2 Experimental section

### 2.1 Cell membrane extraction method

Membrane fractions were isolated from exponentially growing MFC cells at 4°C. Briefly, cells were harvested by centrifugation at 500*g* for 5 min and washed once with ice-cold PBS, and resuspended in ice-cold lysis buffer (50 mM Tris-HCl containing a protease-inhibitor cocktail). After incubation on ice for 1 h, cells were disrupted by probe sonication (2–3s pulses with 10s cooling intervals; total sonication time 10 min). The lysate was clarified by centrifugation at 8000 g for 10 min, and the supernatant was subjected to ultracentrifugation at 100,000 g for 1 h to pellet the membrane-enriched fractions. The resulting membrane pellet was gently resuspended in Tris-HCl buffer, aliquoted, and stored at −80°C. Protein concentrations were determined using the bicinchoninic acid (BCA) assay according to the manufacturer's protocol.

### 2.2 Immunofluorescence staining

The prepared MFC cells were fixed with 4% PFA at 37°C for 20 min, and then treated with 0.01% Triton X-100 for 15 min. Afterwards, the cells were blocked with BSA at room temperature for 1 h. The cells were incubated with the primary antibody overnight at 4°C, followed by incubation with fluorescent secondary antibody at 37°C for 2 h. The nuclei were stained with DAPI for 5 min. Cell images were observed using a laser confocal microscope.

### 2.3 Radiolabeling experiments

For radiolabelling, Iodogen was weighed and dissolved in chloroform within an EP tube. The solvent was evaporated under a controlled nitrogen flow with slow rotation, yielding a uniform Iodogen film on the tube wall. The suspension of Lip^RM^ and Na^131^I solution were sequentially added into the Iodogen-coated reaction tube to initiate the labeling reaction. The mixture was then incubated at 250 rpm at room temperature for 30 min for sufficient reaction. After that, the mixture was centrifuged, and the reaction solution was carefully transferred from the Iodogen-coated tube into a clean tube, as the crude radiolabeled product. Purification was performed using a 100 kDa ultrafiltration tube at 4500 rpm, with repeated ultrafiltration until the radioactivity of the filtrate decreased proportionally after each cycle, yielding the purified product. The radiolabeling efficiency was calculated as the ratio of the radioactivity of the final purified product to that of the crude product.

### 2.4 *In vivo* experiments

For *in vivo* fluorescence imaging, nude mice bearing MFC tumors were intravenously (i.v.) injected with Cy5.5-labeled Lip^RM^ (200 μL, 2 mg/mL), and serial fluorescence images were acquired at different time points using the IVIS system. Separately, for SPECT/CT imaging, MFC tumor-bearing 615 mice were intravenously injected with ^125^I-Lip^RM^ (200 μCi/200 μL), and images were obtained at 1, 6, 12, 24, and 48 h post-injection. For tumor therapy, mice bearing MFC tumors were systematically grouped into 4 groups (G1: Control, G2: Free ^131^I + anti-PD-L1, G3: ^131^I-Lip^R^ + anti-PD-L1, G4: ^131^I-Lip^RM^ + anti-PD-L1). The solid tumors were subjected to H&E, Ki67, and TUNEL staining. The body weight and final tumor volume of the mice were recorded every other day.

### 2.5 Transcriptomics analysis

When the tumors reached approximately 120 mm^3^, the tumor-bearing mice were randomly divided into control group and ^131^I-Lip^RM^ treated group. Following radionuclide therapy, anti-PD-L1 was administered on days 1, 3, and 5. Following completion of the experiments, the tumors were subjected to systematic transcriptomic analysis.

### 2.6 Statistical analyses

In this study, all data are presented as the mean ± SD, and statistical analysis was performed using GraphPad Prism software. The statistical difference between two groups was determined by the *t*-test, and the statistical difference between more than two groups was assessed using two-way ANOVA. P-value < 0.05 was considered statistically significant.

## 3 Results and discussion

### 3.1 Lip^RM^ synthesis and characterization

**Fig. 1:**
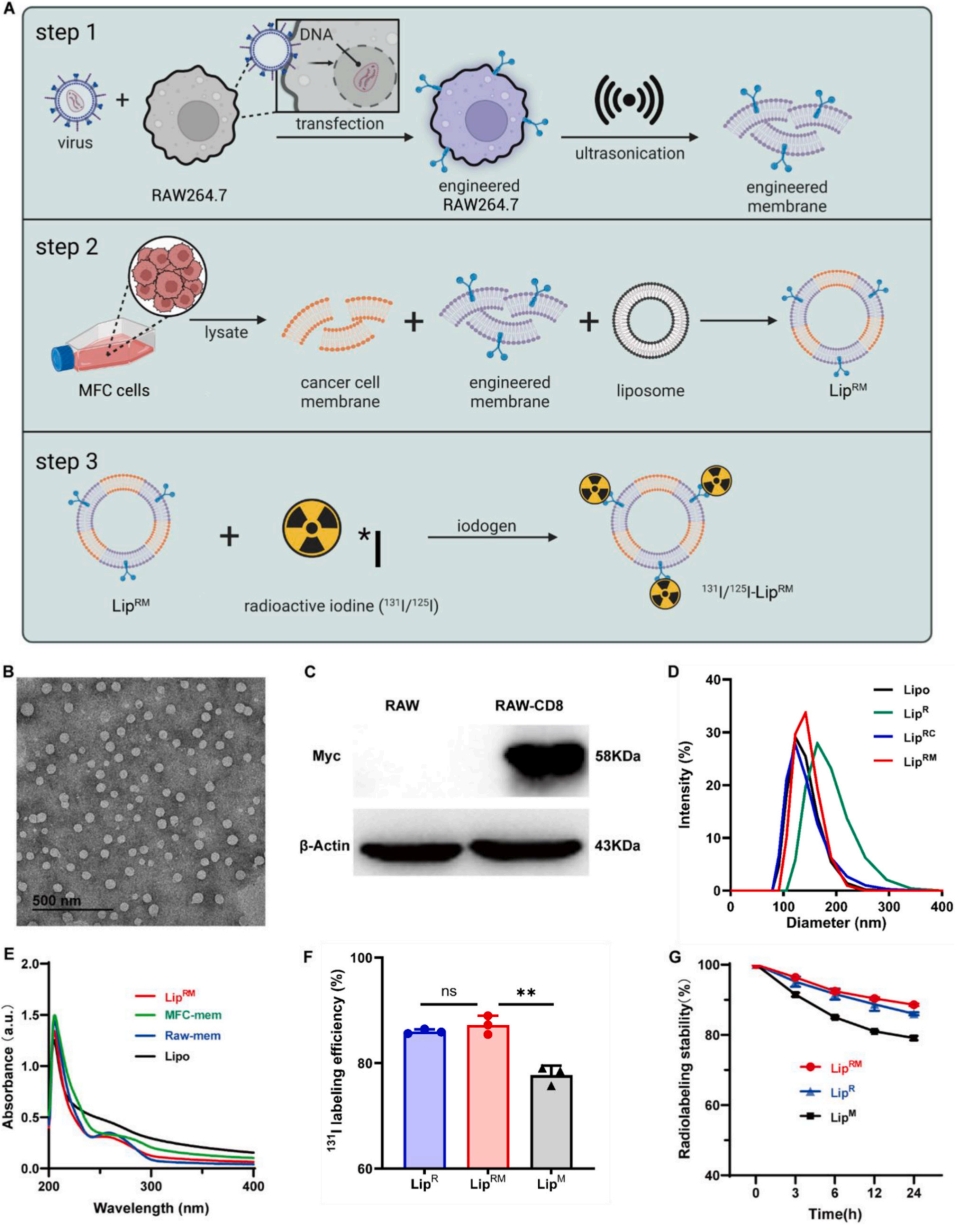
Schematic illustration and characterization of Lip^RM^. **(A)** Schematic synthesis process of the biomimetic Lip^RM^. **(B)** TEM images of Lip^RM^. **(C)** Western blot analysis confirming high expression of Myc-tagged STATH protein in engineered RAW-STATH cells. **(D)** Particle size distribution of Lipo, Lip^R^, Lip^RC^ and Lip^RM^. **(E)** UV-vis absorption spectra of Lipo, MFC-membrane, RAW-membrane, and Lip^RM^. **(F)** Radiolabeling efficiency of different nanoparticle formulations (**p < 0.01, n = 3). **(G)** Radiolabeling stability of various formulations, showing enhanced labeling efficiency and stability using hybrid RAW-STATH cell membranes.

To achieve targeted radionuclide delivery and modulate the immune microenvironment for enhanced tumor immunotherapy, we constructed Lip^RM^, a radio-labelable hybrid cell membrane biomimetic vector (Fig. 1A). RAW264.7 cells were transfected with a plasmid to achieve high surface expression of the tyrosine-rich protein Statherin (STATH) to potentiate immune response and enhance radioiodination efficiency. Specifically, the lentiviral vector pLVX was constructed to encode a fusion protein consisting of a CD8 peptide sequence (hinge domain, serving as a membrane anchor), the tyrosine-rich STATH sequence, and a C-terminal Myc tag for detection (Fig. S1). Scanning electron microscopy (SEM) revealed that bare liposomes (Lipo) exhibited a spherical morphology with an average diameter of approximately 120 nm (Fig. S2). In contrast, SEM and TEM imaging of Lip^RM^ (Fig. S3, Fig. 1B) showed larger particles with an average diameter of around 130 nm. The distinct bilayer-like structure observed in TEM further confirmed successful membrane coating, which is critical for the functional mimicry of native cells. Western blot analysis (Fig. 1C) confirmed successful engineering, evidenced by substantial Myc tag expression in the modified RAW-CD8 cells, thereby validating the expression of the fusion protein required for downstream membrane extraction. In addition, the particle sizes of Lipo and Lip^RM^ were detected by dynamic light scattering (DLS). Notably, the Lip^RM^ exhibited a size peak in the range of approximately 130-150 nm (Fig. 1D), which falls within the optimal range for enhanced permeability and retention (EPR) effects, suggesting the potential for effective passive tumor targeting. This size increase compared to bare Lipo also implied successful membrane coating, which contributed to the biological stealth and targeting properties of the nanoparticles. Zeta potential measurements (Fig. S4A) showed a significant difference between Lipo and Lip^RM^ (p < 0.01, n = 3), indicating marked changes in surface charge upon membrane fusion. The reduced absolute zeta potential of Lip^RM^ might be attributed to the incorporation of natural membrane components that partially neutralized the cationic surface of the liposomes. Such surface charge modulation was essential not only for improving colloidal stability in physiological environments but also for enhancing immune evasion and prolonging systemic circulation. Consistent with previous reports, the altered zeta potential reflects successful membrane cloaking and functional transformation of the liposomal surface [38].

Furthermore, UV-vis absorbance spectra (Fig. 1E) of Lipo, MFC membrane (MFC-mem), RAW264.7 membrane (RAW-mem), and Lip^RM^ exhibited distinct optical features, reflecting the compositional differences and successful integration of membrane materials. We compared the radiolabeling efficiency of the different nanoparticle formulations (Fig. 1F). Lip^RM^ exhibited the highest ^131^I labeling efficiency, which was significantly greater than that of Lip^M^, while no significant difference was observed between Lip^R^ and Lip^RM^. Moreover, radiolabeling stability was improved in the hybrid membrane-coated system. ^131^I-Lip^RM^ maintained the highest radioactivity retention at each time point from 0 to 24 h in PBS (Figs. 1G) and 20% FBS (Fig. S5A), indicating that the engineered RAW-MFC membrane enhanced both iodine binding and retention. These results indicated that fusing liposomes with RAW and MFC cell membranes could enhance ^131^I labeling efficiency and improves radiolabel stability in vitro (Fig. S5B), suggesting potential advantages for the stability of radiotracers and their *in vivo* tracing performance. Collectively, these results validated the successful construction of Lip^RM^ with distinct physicochemical and structural characteristics, laying a solid foundation for their application in targeted radionuclide delivery and immunotherapeutic modulation.

### 3.2 Cellular-level modulation of radionuclide-labeled Lip^RM^

**Fig. 2:**
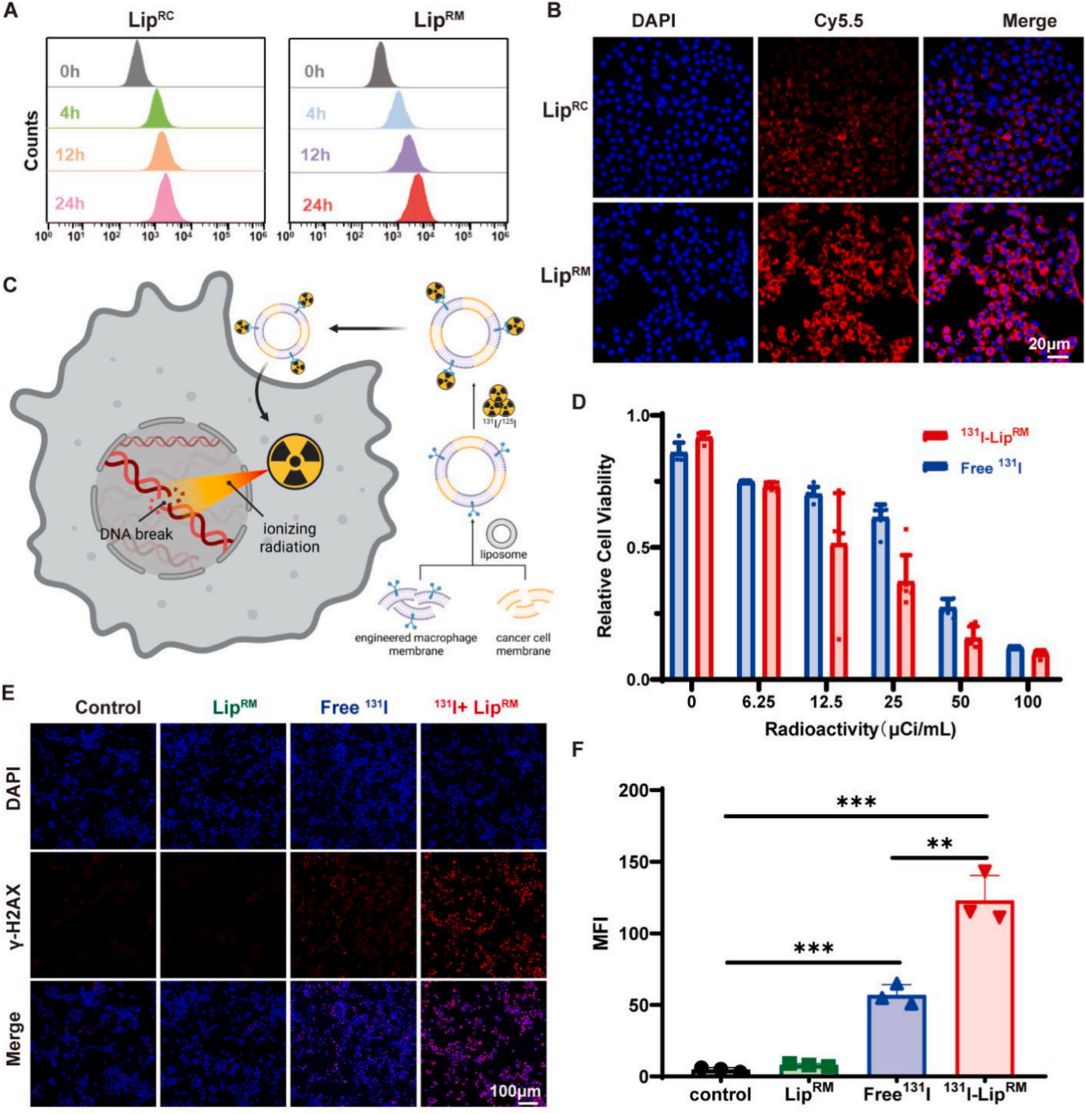
Cellular evaluation of ^131^I-Lip^RM^. **(A)** Flow cytometry analysis of nanoparticle uptake by MFC cells. **(B)** Confocal microscopy images of MFC cells incubated with different biomimetic nanoparticles for 24 h (Cy5.5-labeled), confirming enhanced homologous targeting at the cellular level. **(C)** Schematic illustration of the proposed cellular killing mechanism. **(D)** Relative viability of MFC cells treated with different doses of ^131^I-labeled nanoparticles for 24 h(n = 4). **(E, F)** Confocal microscopy images of MFC cells 24 h post-treatment under different conditions (blue: DAPI-stained nuclei; red: γ-H2AX indicating DNA damage, n = 3, **p < 0.01, ***p < 0.001).

After evaluating the physicochemical properties and functionalities of the hybrid membrane nanoparticles, we further assessed their regulatory effects and therapeutic efficacy at the cellular level following radionuclide labeling. Flow cytometry analysis demonstrated markedly increased cellular uptake of the hybrid membrane-coated Lip^RM^ compared to hybrid controls (Lip^RC^),which derived from fusing liposome with statherin engineered macrophage membranes and colon cancer cell (CT-26) membranes (Fig. 2A). Furthermore, confocal laser scanning microscopy of MFC cells incubated with different nanoparticle formulations for 24 h showed enhanced cellular uptake of Lip^RM^ compared to Lip^RC^, as evidenced by stronger Cy5.5 fluorescence (Fig. 2B). A schematic diagram illustrated the proposed cellular killing mechanism, which involved radiation-induced DNA damage synergized by targeted nanoparticle delivery (Fig. 2C). Upon labeling with ^131^I, the nanoparticles exhibited significantly enhanced cytotoxicity (Fig. 2D), confirming the effective radiotherapeutic action of the radionuclide payload. Furthermore, Lip^RM^ without ^131^I labeling exhibited no significant cytotoxicity to MFC gastric cancer cells after 24 h of incubation (Fig. S7). Importantly, confocal laser scanning microscopy revealed elevated levels of γ-H2AX—a specific marker of DNA double-strand breaks—in MFC cells treated with ^131^I-labeled Lip^RM^ (Fig. 2E and F). Collectively, these findings demonstrated that ^131^I-Lip^RM^ not only possessed excellent tumor-targeting and uptake capabilities but also achieved efficient and stable radionuclide labeling, thereby eliciting potent therapeutic effects at the cellular level and establishing a solid foundation for future *in vivo* applications.

### 3.3 *In vivo* tumor targeting of radiolabeled Lip^RM^

**Fig. 3:**
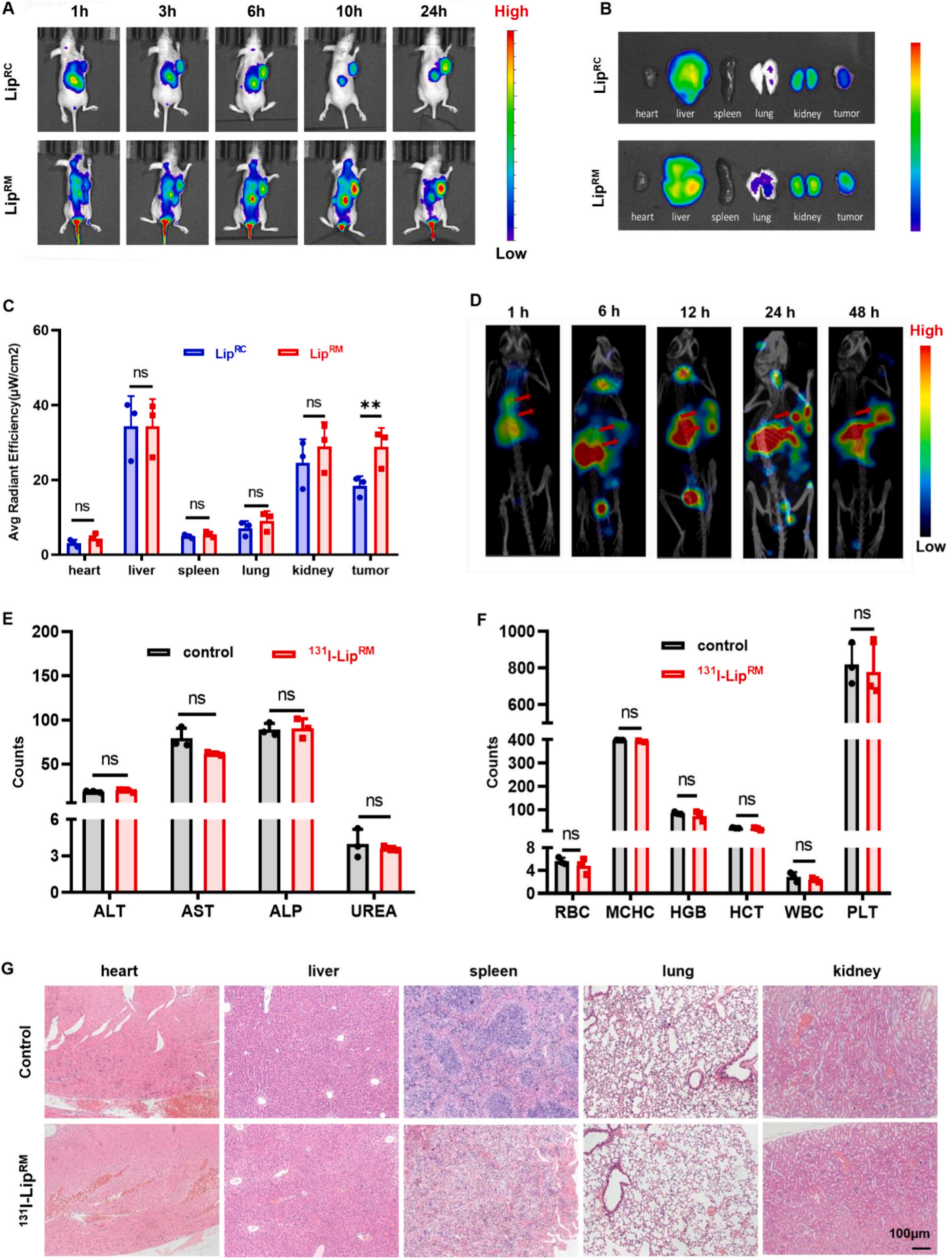
Homologous tumor-targeting capability of Lip^RM^*in vivo* and in vitro. **(A)** IVIS imaging of MFC subcutaneous tumor-bearing nude mice showed enhanced tumor accumulation in the Lip^RM^ group compared to the control group. **(B)***Ex vivo* IVIS imaging of major organs at 24 h post-injection, comparing targeting performance between groups. **(C)** Quantification of fluorescence intensity in major organs 24 h after injection (**p < 0.01, n = 3). **(D)** SPECT-CT reconstructions at multiple time points after injection of biomimetic nanoparticles demonstrated precise tumor localization. **(E, F)** Hematological parameters and liver/kidney function indices at 24 h after intravenous injection of Lip^RM^, indicating no significant systemic toxicity. **(G)** H&E staining of major organs (heart, liver, spleen, lung, kidney) following treatment with radionuclide-labeled nanoparticles. Data are presented as mean ± SD (n = 3); **p < 0.01.

Building on the demonstrated *in vivo* tumor targeting and biodistribution of Lip^RM^, we next compared the homologous targeting efficiency of Lip^RM^ and Lip^RC^. In the subcutaneous tumor-bearing nude mouse model, the results showed that Lip^RM^ exhibited significantly enhanced tumor accumulation compared with Lip^RC^ group (Fig. 3A). This suggested that incorporation of MFC-derived membrane components endowed the nanoparticles with tumor-recognition capabilities via homotypic binding, a feature commonly observed in cell membrane-coated delivery systems. *Ex vivo* IVIS imaging of major organs at 24 h after intravenous injection further confirmed that the fluorescence signal in the tumor was markedly higher in the Lip^RM^ treated group than in the control group (Fig. 3B). Quantitative analysis of the fluorescence intensity from these organs revealed statistically significant increases in tumor uptake in the Lip^RM^ treated group (p < 0.01, n = 3), while signals in other organs showed no significant differences (Fig. 3C). These results indicate that the hybrid membrane coating promotes tumor homing, while partial hepatic and renal uptake reflects typical clearance routes for nanoscale carriers.

Meanwhile, we performed biodistribution analysis using ^125^I-Lip^RM^. At 24 h post-injection, gamma counting demonstrated selective accumulation in the tumor, with uptake reaching 2.64 ± 1.84% ID/g, as well as notable distribution in the liver and kidneys (Fig. S9). In addition, serial SPECT/CT imaging was performed to monitor dynamic *in vivo* distribution of ^125^I-Lip^RM^, revealing time-dependent enrichment of radioactivity within the tumor region (Fig. 3D). Collectively, these results confirmed that Lip^RM^ exhibited robust *in vivo* tumor-targeting performance, effective radiolabeling retention, and favorable biodistribution, highlighting its potential as a targeted delivery platform for radionuclide-based cancer therapy. To evaluate the biosafety profile of the ^131^I-Lip^RM^, comprehensive assessments were conducted during and after treatment. Given that changes in mouse behavior and body weight are indicative of acute toxicity, and macroscopic pathological alterations reflect potential long-term toxicity, these parameters were closely monitored throughout the study [39,40]. Hematological and biochemical analyses performed 24 h after intravenous administration of ^131^I-Lip^RM^ revealed no significant abnormalities in routine blood parameters or liver and kidney function markers, suggesting minimal acute systemic toxicity (Fig. 3E and F). These findings were consistent with the expected low toxicity of biomimetic nanoparticles, which often exhibited enhanced biocompatibility due to their membrane-coated nature, reducing the likelihood of immune system recognition and clearance. Furthermore, histopathological examination of major organs (heart, liver, spleen, lung, and kidney) following combined treatment with radiolabeled nanoparticles showed no observable tissue damage or inflammatory infiltration across all groups, indicating good long-term biocompatibility (Fig. 3G). The lack of organ-specific toxicity or inflammation further supported the notion that the hybrid membrane coating mitigates off-target effects, potentially reducing the side effects commonly associated with conventional therapeutic nanoparticles or radionuclide therapies.

### 3.4 Therapeutic and immune response evaluation of ^131^I-Lip^RM^*in vivo*

**Fig. 4:**
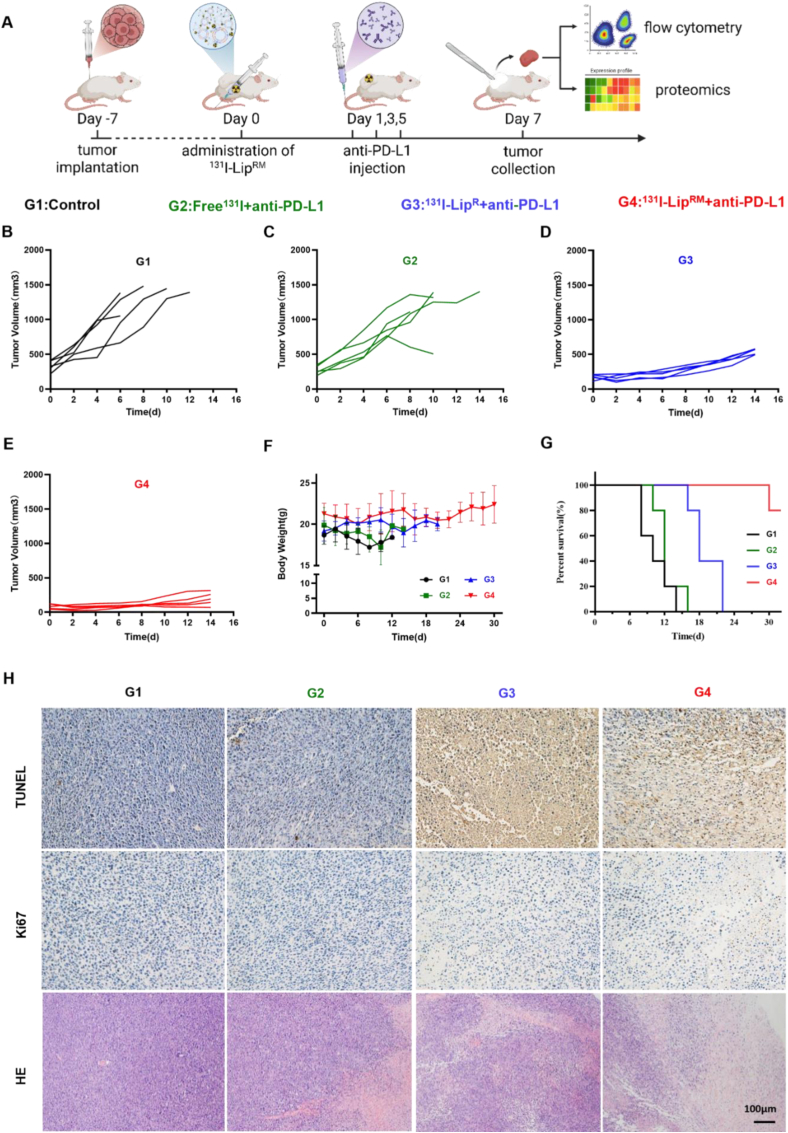
*In vivo* antitumor efficacy of ^131^I-Lip^RM^. **(A)** Schematic illustration of the treatment protocol for evaluating therapeutic efficacy in the subcutaneous MFC tumor model. **(B-E)** Tumor growth curves showing significant tumor inhibition in the group treated with ^131^I-Lip^RM^. **(F)** Body weight changes of mice during treatment, indicating no significant toxicity across all groups. **(G)** Kaplan-Meier survival curves demonstrating prolonged survival in the ^131^I-Lip^RM^ group. **(H)** Histological analysis of tumor sections by H&E staining, Ki67 immuno-histochemistry, and TUNEL assay, showing enhanced apoptosis and reduced proliferation in the ^131^I-Lip^RM^ group. Data are presented as mean ± SD (n = 5); **p < 0.01. G1 (control), G2 (Free 131I + anti-PD-L1), G3 (131I-Lip^R^ + anti-PD-L1), and G4 (131I-Lip^RM^ + anti-PD-L1).

To further evaluate the therapeutic potential of ^131^I-Lip^RM^ *in vivo*, a subcutaneous MFC tumor model was established in mice. When the tumor volume reached approximately 120 mm^3^, the mice were randomly divided into four groups: G1 (control), G2 (Free ^131^I + anti-PD-L1), G3 (^131^I-Lip^R^ + anti-PD-L1), and G4 (^131^I-Lip^RM^ + anti-PD-L1). As illustrated in the treatment schematic (Fig. 4A), an *in vivo* therapeutic study was conducted to evaluate the efficacy of ^131^I-Lip^RM^ + anti-PD-L1. Tumor growth curves showed that the group treated with G4 exhibited the most significant tumor growth inhibition compared to the other groups (Fig. 4B–E), indicating superior therapeutic performance of the biomimetic formulation. This enhanced efficacy likely stemmed from the combination of targeted radionuclide delivery and immune microenvironment modulation conferred by the dual-source membrane coating. Throughout the treatment period, no notable body weight loss was observed among the groups, suggesting good systemic biocompatibility and safety (Fig. 4F). Survival analysis further demonstrated a marked extension of overall survival in the G4 group (Fig. 4G), supporting the long-term benefit of the treatment. These findings were consistent with visual tumor comparisons and tumor weight measurements at the endpoint, both of which showed significantly reduced tumor burden in this group (Fig. S10–S11). Histological examinations, including TUNEL, Ki-67, and H&E staining, revealed enhanced apoptosis, reduced proliferation, and substantial tumor tissue destruction (Fig. 4H), further validating the therapeutic benefits of the treatment at the cellular and tissue levels. These results confirm that the nanoparticles effectively deliver radionuclide payloads and induce robust tumor cell death.

### 3.5 Immunomodulatory effects of ^131^I-Lip^RM^ + anti-PD-L1

**Fig. 5:**
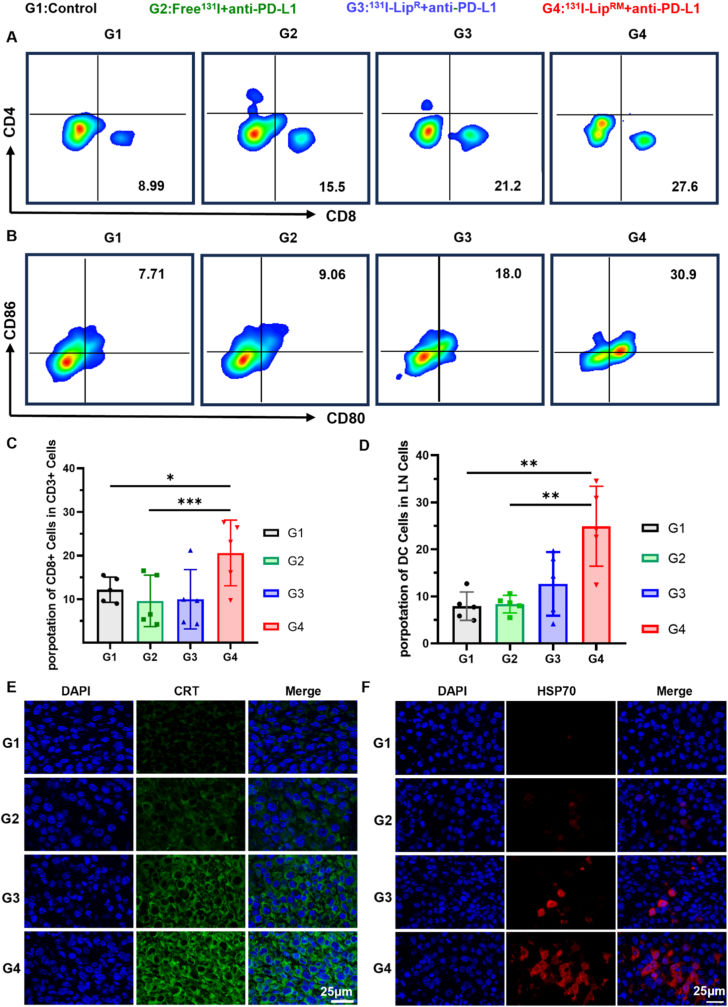
Flow cytometry analysis of immune cells in the tumor microenvironment. **(A, C)** CD8^+^ T cell levels in control and ^131^I-Lip^RM^ + anti-PD-L1 groups. **(B, D)** Mature dendritic cells in the same groups. **(E, F)** Immunofluorescence staining of CRT and HSP70 revealed elevated expression of immunogenic cell death markers in treated tumors. Data are presented as mean ± SD (n = 5); *P < 0.05, **P < 0.01, ***P < 0.001.

To further evaluate the immune activation induced by ^131^I-labeled Lip^RM^, we performed flow cytometry to assess CD8^+^ cytotoxic T lymphocytes and dendritic cell (DC) populations in the tumor microenvironment. Treatment with ^131^I-Lip^RM^ + anti-PD-L1 led to a significant increase in CD8^+^ T cells (Fig. 5A–C) and mature DCs (Fig. 5B–D) compared to the control group. Notably, the proportion of DCs increased from 7.91 ± 3.02% in the control group to 24.92 ± 8.52% following treatment, indicating a substantial enhancement of antigen-presenting capacity. This elevation in DC maturation suggested that the formulation not only enhanced radionuclide delivery but also stimulated local immune activation. The hybrid RAW-MFC membrane, likely providing both tumor-associated antigens and immunostimulatory components, facilitates DC maturation and promotes CD8^+^ T cell recruitment, enhancing the overall antitumor immune response. Moreover, immunofluorescence staining of CRT and heat shock protein 70 (HSP70) demonstrated markedly elevated CRT exposure and HSP70 expression in the G4 group, indicating that the treatment not only induced tumor cell death but also promoted ICD, which may further facilitate antitumor immune responses (Fig. 5E and F). Collectively, these results underscore the strong potential of ^131^I-Lip^RM^ + anti-PD-L1 for effective radionuclide-based cancer therapy, with the added benefit of promoting anti-tumor immune activation.

It should be noted that the enhanced CD8^+^ T-cell infiltration following ^131^I-Lip^RM^ plus anti-PD-L1 therapy was likely driven by multiple interrelated mechanisms. Beyond immunogenic cell death (ICD)-associated antigen release and dendritic cell maturation, radiation-induced vascular permeability and local inflammatory responses may also facilitate immune cell extravasation and recruitment into the tumor microenvironment. Therefore, the observed increase in CD8^+^ T-cell infiltration should be interpreted as the integrated outcome of both active antitumor immune activation and radiation-mediated physical and inflammatory remodeling of the tumor milieu. This multifactorial view was further supported by our transcriptomic and proteomic data, which showed concurrent enrichment of immune activation, antigen processing, phagosome formation, and inflammatory signaling pathways.

### 3.6 Multi-omics verification of the Immunomodulatory Capabilities

**Fig. 6:**
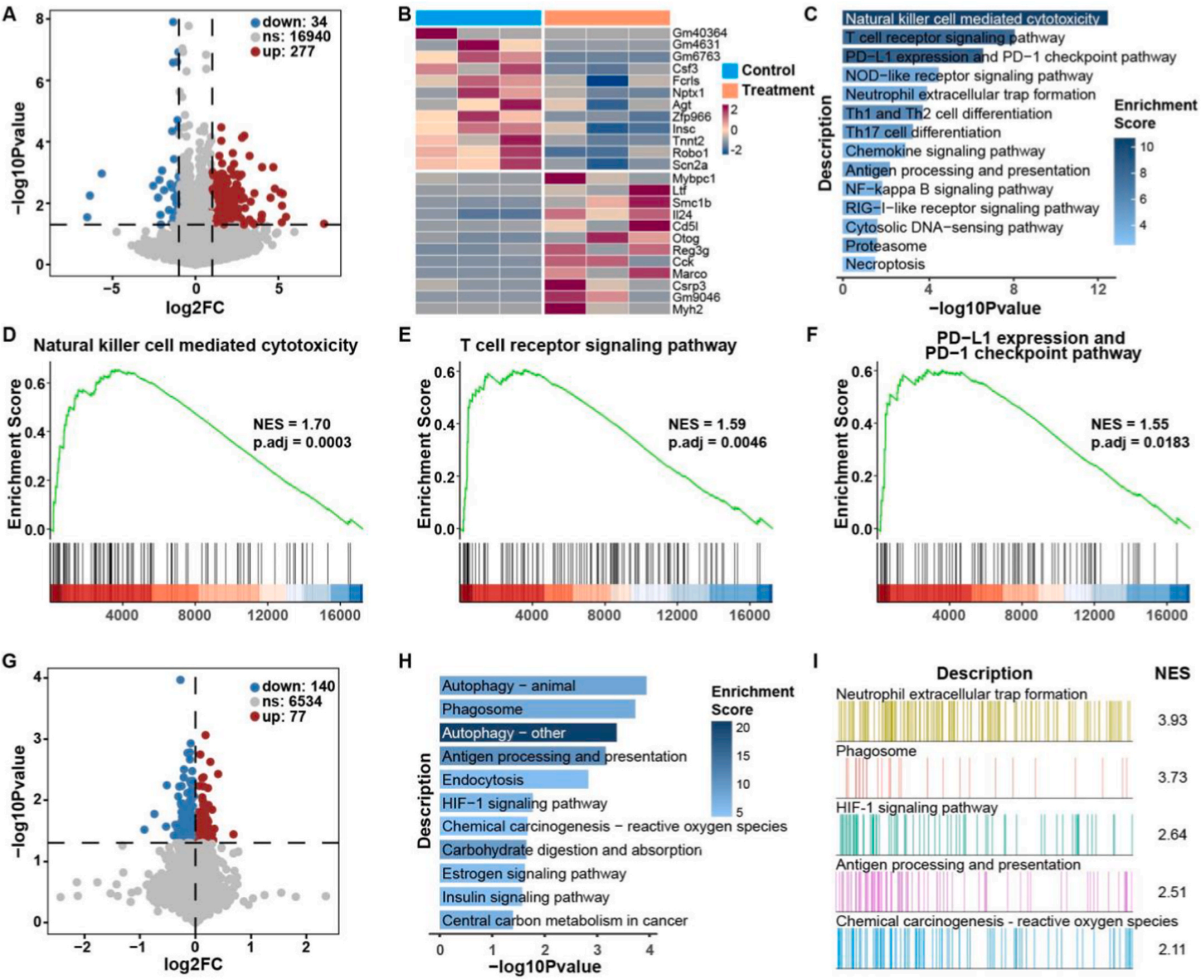
Multi-omics Verification of the Immunomodulatory Capabilities. **(A, B)** Volcano plot of differentially expressed genes (DEGs) between control and treated groups. **(C)** KEGG pathway enrichment indicating involvement of immune- and cancer-related pathways. **(D-F)** GSEA enrichment plots for immune effector pathways. **(G)** Volcano plot of protein expression changes between treated and control tumor samples. **(H)** KEGG pathway enrichment of differentially expressed proteins. **(I)** Representative GSEA enrichment plots for selected protein-level gene sets; the enrichment score curve is shown with vertical tick marks indicating gene positions in the ranked list; normalized enrichment scores (NES) and adjusted P values are reported on each plot.

Transcriptomics is a powerful tool for comprehensively analyzing global gene expression changes and understanding molecular mechanisms underlying disease progression and therapeutic responses [41,42]. To investigate the gene expression alterations induced by ^131^I-Lip^RM^, we performed a comparative analysis of differentially expressed genes (DEGs) between the control and ^131^I-Lip^RM^ + anti-PD-L1 groups (Fig. 6A). A total of 17,251 genes were identified across both groups, among these, 311 genes were found to be significantly regulated following treatment, representing key targets modulated by the biomimetic nanoparticle system (Fig. 6B).

To further elucidate the therapeutic mechanisms of ^131^I-Lip^RM^, KEGG enrichment analysis was performed based on DEGs. KEGG analysis revealed significant enrichment in multiple immune- and cancer-related pathways, including T cell receptor signaling, TGF-β signaling, NK cell-mediated cytotoxicity, NF-κB signaling, and PD-1 checkpoint regulation. These findings indicated that immune activation and inflammatory responses are integral to the antitumor mechanism (Fig. 6C). Consistent with these results, GSEA demonstrated positive enrichment of immune effector signatures, specifically, natural killer cell-mediated cytotoxicity (NES = 1.70, p = 0.0003), T-cell receptor signaling (NES = 1.59, p = 0.0046), and PD-L1/PD-1 checkpoint pathway (NES = 1.55, p = 0.0183) (Fig. 6D–F). Collectively, these data suggested that ^131^I-Lip^RM^ exerted antitumor effects through multifaceted modulation of immune signaling and transcriptional regulation within the tumor microenvironment.

To investigate the mechanisms at the protein level, we conducted a comparative proteomic analysis between treated and control tumors. This analysis identified 217 differentially expressed proteins (77 upregulated, 140 downregulated) (Fig. 6G). Pathway enrichment highlighted autophagy and phagosome processes, antigen processing and presentation, endocytosis, and HIF-1 signaling, alongside pathways related to oxidative stress and metabolism (Fig. 6H). Protein-level GSEA corroborated these findings, showing strong enrichment for neutrophil extracellular trap formation (NES = 3.93), phagosome (NES = 3.73), HIF-1 signaling (NES = 2.64), antigen processing and presentation (NES = 2.51), and chemical carcinogenesis-reactive oxygen species (NES = 2.11) (Fig. 6I). Overall, the proteomic profile indicated coordinated modulation of autophagy/phagocytic pathways, antigen-presentation machinery, and hypoxia/oxidative-stress-related metabolism, consistent with altered innate immune and stress responses and likely contributing to the observed antitumor efficacy.

Mechanistically, the observed alterations in gene and protein expression reflect the integrated consequences of direct radiobiological effects and subsequent immune activation. First, the enrichment of differentially expressed proteins in the “HIF-1 signaling” and “Reactive oxygen species” pathways (Fig. 6H and I) represents a canonical cellular response to ionizing radiation. The β-particles emitted by ^131^I drive water radiolysis, generating excessive ROS that overwhelm cellular antioxidant capacity, thereby inducing oxidative damage and hypoxic stress responses. Second, and critically, the upregulation of “Antigen processing and presentation” (Fig. 6H) and “T cell receptor signaling” (Fig. 6C) provides direct molecular evidence of radiation-induced immunogenic modulation. In particular, radiation-triggered immunogenic cell death (ICD) expands the availability of tumor antigens, while the coordinated upregulation of antigen-presenting machinery suggests an enhanced capacity of dendritic cells to prime naïve T cells. Collectively, these molecular shifts convert the tumor microenvironment into an immunologically “hot” state, which underpins the observed CD8^+^ T cell infiltration (Fig. 5A) and provides a mechanistic basis for the synergistic efficacy when combined with immune checkpoint blockade.

While our preclinical results are promising, it is crucial to contextualize this work within the current landscape of clinical radionuclide therapy for gastric cancer. Currently, systemic radionuclide therapies (such as radioimmunotherapy) have shown potential but remain challenging for gastric adenocarcinoma due to tumor heterogeneity [43]. Conventional strategies, often relying on antibody-drug conjugates labeled with ^177^Lu or ^131^I (e.g., targeting CLDN18.2), face the “binding-site barrier,” where high-affinity binding to perivascular antigens prevents deep penetration into the tumor mass [44,45]. Furthermore, circulating free radionuclides often lead to dose-limiting toxicities, particularly myelosuppression [45,46]. Our ^131^I-Lip^RM^ strategy offers distinct advantages over these clinical standards. By utilizing a biomimetic nanoplatform, we leverage the Enhanced Permeability and Retention (EPR) effect and active homotypic targeting to bypass the penetration limits of large antibodies, thereby achieving higher intratumoral accumulation. Additionally, the liposomal encapsulation ensures the stability of ^131^I, significantly reducing the off-target toxicity associated with free iodine. However, we must acknowledge the limitations of this nanomedicine approach. Compared to small-molecule inhibitors, nanocarriers may face transport resistance in tumors with extremely high interstitial fluid pressure [47]. Moreover, the complexity of GMP production for hybrid membrane-coated nanoparticles presents a higher threshold for clinical translation than standard radiolabeling procedures. Future studies will focus on optimizing the size and deformability of the nanoparticles to further enhance deep-tissue penetration.

## 4 Conclusion

In this study, we successfully developed a novel, hybrid cell membrane-camouflaged nanoparticle platform, ^131^I-Lip^RM^, for targeted radionuclide delivery and synergistic radio-immunotherapy in gastric cancer. By fusing liposomes with engineered macrophage membranes (rich in tyrosine for efficient radiolabeling) and homologous gastric cancer cell membranes (MFC), the constructed biomimetic nanoparticles achieved high-yield and stable ^131^I labeling, coupled with enhanced tumor-targeting capability through dual mechanisms: the inflammatory tropism of macrophages and homotypic binding of cancer cell membranes. *In vitro*, ^131^I-labeled Lip^RM^ exhibited strong cytotoxicity and induced DNA damage. *In vivo* imaging and biodistribution studies further confirmed precise tumor targeting with minimal nonspecific uptake. Therapeutic studies in mouse models showed significant tumor inhibition and prolonged survival, particularly when combined with anti-PD-L1. Flow cytometry and multi-omics analyses provided supportive evidence for the treatment-induced immune response. Altogether, our findings highlight that the ^131^I-Lip^RM^ platform represents a promising and multifaceted strategy for advanced gastric cancer treatment. It seamlessly integrates precise radionuclide delivery with effective immune activation, offering a potent avenue for synergistic radio-immunotherapy with significant potential for clinical translation.

## Ethics approval and consent to participate

All animal studies were approved by the Animal Ethics and Welfare Committee of Soochow University.

## Disclosure

The authors declare no conflict of interests in this work.

## CRediT authorship contribution statement

**Chuanqiang Zhang:** Formal analysis, Investigation, Methodology, Software, Validation, Visualization, Writing – original draft. **Hua Chen:** Investigation, Methodology. **Zheng Yuan:** Investigation, Methodology. **Anqi Dong:** Investigation, Methodology. **Shu Liu:** Investigation, Methodology, Software, Visualization. **Lin Hu:** Conceptualization, Supervision, Writing – review & editing. **Kai Yang:** Conceptualization, Funding acquisition, Writing – original draft, Writing – review & editing. **Jin Zhou:** Conceptualization, Data curation, Funding acquisition, Project administration, Writing – review & editing.

## Declaration of competing interest

The authors declare that they have no known competing financial interests or personal relationships that could have appeared to influence the work reported in this paper.

## Data Availability

Data will be made available on request.
