# Supplementary material for: 131I-labeled engineered hybrid cell membrane biomimetic nanocarrier targets gastric cancer and enhances radioimmunotherapy
Source: Mater Today Bio. 2026 Aug 4;40:103533. doi: 10.1016/j.mtbio.2026.103533 (PMC13475372; doi:10.1016/j.mtbio.2026.103533)
Supplement: Supplementary file 1 [file mmc1.docx]

**¹³¹I-Labeled Engineered Hybrid Cell Membrane Biomimetic Nanocarrier Targets Gastric Cancer and Enhances Radioimmunotherapy**

Chuanqiang Zhang^1, 2#^, Hua Chen^3#^, Zheng Yuan^1^, Anqi Dong^3^, Shu Liu^3^, Lin Hu^3^, Kai Yang^3^, Jin Zhou^1^
^1^ Department of General Surgery, The First Affiliated Hospital of Soochow University, Suzhou, 215006, Jiangsu, People’s Republic of China
^2^ Department of General Surgery, The Affiliated Jiangsu Shengze Hospital of Nanjing Medical University, Suzhou, 215228,Jiangsu, People’s Republic of China
^3^ State Key Laboratory of Radiation Medicine and Protection, School of Radiation Medicine and Protection & School for Radiological and Interdisciplinary Sciences (RAD-X), Collaborative Innovation Center of Radiation Medicine of Jiangsu Higher Education Institutions, Soochow University, Suzhou, Jiangsu 215123, People’s Republic of China
#Contributed equally to this work

Corresponding Author Email: kyang@suda.edu.cn, [zhoujinsuda@suda.edu.cn](mailto:zhoujinsuda@suda.edu.cn)

**Materials**

Glutathione (GSH,70-18-8) and sodium borohydride (NaBH4, 16940-66-2) were purchased from Sigma-Aldrich. Tetrachloroauric acid tetrahydrate (HAuCl4·4H2O, 16903-35-8), sodium hydroxide (NaOH, 1310-73-2), hydrochloric acid (HCl, 7647-01-0), and dimethyl sulfoxide (DMSO, 67-68-5) were obtained from Sinopharm Chemical Reagent Co., Ltd., Suzhou. Cell culture medium (Hyclone) and fetal bovine serum (Gibco), along with other cell culture supplies were all purchased from Suzhou Yibon Biotechnology Co., Ltd. The Cy5.5 NHS ester was procured from Shandong Sparkjade Biotechnology Co., Ltd. The compound Iodogen was procured from Aladdin. Na^125^I and Na^131^I solutions were procured from Shanghai GMS Pharmaceutical Co. The γ-H2AX antibody and flow cytometry detection antibodies were obtained from eBioscience.

**Characterization**

Elemental analysis was performed using an ICAP7200 inductively coupled plasma optical emission spectrometer. Imaging studies utilized a U-SPECT^+^ small animal SPECT-CT imaging system, an Olympus FV1200 scanning laser confocal microscope. Radioactivity measurements were conducted with an FJ-391A4 radioactivity dose calibrator. Spectroscopy analysis included a UV-3600 UV-Vis-NIR spectrophotometer and an FLS980 steady-state and transient fluorescence spectrometer. Nanoparticle size and charge were assessed with a Zetasizer Nano ZS90 analyzer.

**Cell Lines and Animals**

CT26 colorectal cancer cells, MFC gastric cancer cells, and RAW264.7 macrophages were obtained from the Chinese Academy of Sciences Cell Centre in Shanghai and cultured under standard laboratory conditions. BALB/c-nude, aged 6-8 weeks, were purchased from Nanjing Junke Bioengineering Co., Ltd; 615 mice with a fully functional immune system were purchased from Shanghai Sipple-Bikai Laboratory Animal Co., Ltd. The animal experiments were conducted in accordance with the ethical standards set by the Experimental Animal Center of Soochow University. To establish the subcutaneous tumor model, approximately 2 × 10^6^ MFC cells suspended in 50 μL of PBS were injected subcutaneously into the dorsal region of the mice.

***In vitro* Cytotoxicity Assay**

The CCK8 assay was used to determine the cytotoxicity of MFC cells. According to the experimental requirements, MFC cells were cultured in a 96-well plate. Different concentrations of nanoparticles were then added to each well and incubated for 24 h. Three parallel samples were set for each group, followed by the addition of 10 µL CCK-8 to each well and incubation for 4 h. The absorbance was quantified at 450 nm using a spectrophotometer.

**Cellular Uptake Analysis**

The nanoparticles were co-incubated with MFC cells for different time periods. The cells were then washed with PBS, and the BD FACSVerse was used to read the cells. FlowJo software was used to analyze the uptake.

**Figure S1.** Plasmid design: the pLVX vector encodes a CD8 hinge domain fused with a tyrosine-rich STATH sequence and a C-terminal Myc tag.

**Figure S2.** SEM image of bare liposomes.

**Figure S3.** SEM image of Lip^RM^ nanoparticles.

**Figure S4. (A)** Zeta potential of Lipo, Lip^R^, Lip^RC^ and Lip^RM^ (n=3, **p<0.01). **(B)** Coomassie blue staining results of Lipo, Lip^R^, Lip^RC^ and Lip^RM^.

**Figure S5. (A) Representative radio-TLC results of ^131^I-Lip^R^, ^131^I-Lip^M^, and ^131^I-Lip^RM^ after radiolabeling. Samples were spotted 1 cm from the bottom edge of the TLC plate, and normal saline was used as the mobile phase. (B) Radio-TLC results of post-ultrafiltration ^131^I-Lip^R^, ^131^I-Lip^M^, and ^131^I-Lip^RM^ incubated in 20% FBS at 37°C for 1, 3, 6, 12, and 24 h. The radiolabeled nanoparticle fraction and released free ^131^I fraction were quantified using Laura radio-TLC software. Data are presented as mean ± SD, n = 3.**

**Figure S6.** Confocal microscopy images of SNU-1 cells incubated with different biomimetic nanoparticles for 24 h (Cy5.5-labeled), confirming enhanced homologous targeting at the cellular level.

**Figure S7.** Relative viability of MFC cells incubated with varying concentrations of nanoparticles for 24 h (n=3).

**Figure S8.** IVIS imaging of MFC subcutaneous tumor-bearing nude mice showed enhanced tumor accumulation in the Lip^R^ group compared to the Lip^M^ group.

**Figure S9. (A)** Biodistribution of ^125^I-Lip^RM^ in mice 24 h after injection (n=3). **(B)** ROI-based tumor/muscle radioactivity count ratio calculated from serial SPECT/CT images within 48 h after injection. **(C)** ROI-based tumor/liver radioactivity count ratio calculated from serial SPECT/CT images within 48 h after injection.

**Figure S10.** Representative images of tumors collected at the endpoint.

**Figure S11. Final tumor volumes at study endpoint. Data are mean ± SD (n=5, *P < 0.05, ***P < 0.001).**
